# Heavy Metal Air Pollution: Human Health Effects and Nanomaterial Strategies for Monitoring and Remediation

**DOI:** 10.1007/s12011-026-05019-3

**Published:** 2026-02-23

**Authors:** Yosri A. Fahim, Haneen M. Boughdady, Walid A. Othman, Sondos M. Mostafa, Lara M. Helmi, Ahmed A. Elsayed, Omar S. Hamouda, Osama M. Gad, Reem M. Sallam

**Affiliations:** 1 Department of Basic Medical Sciences, Faculty of Medicine, Galala University, Suez, 43511 Egypt; 2 Faculty of Medicine, Galala University, Suez, 43511 Egypt; 3 Department of Medical Biochemistry & Molecular Biology, Faculty of Medicine, Ain Shams University, Cairo, Egypt

**Keywords:** Heavy metal, Human health risks, Systemic diseases, Nanotechnology, Nanomaterials

## Abstract

Heavy metal air pollution represents a critical global environmental and public health challenge due to its persistence, bioaccumulation, and diverse toxic effects. Lead, mercury, cadmium, and arsenic are among the most hazardous, disrupting biological systems through oxidative stress, chronic inflammation, and interference with cellular signaling. These mechanisms drive the onset of acute and chronic diseases, damaging multiple organ systems and reducing overall health quality. The respiratory system is highly susceptible, as inhalation of fine and ultrafine particulates induces oxidative injury, inflammation, fibrosis, and progressive loss of lung function. Cardiovascular disorders such as endothelial dysfunction, hypertension, and atherosclerosis arise from oxidative stress and calcium signaling disruption, while endocrine toxicity occurs when heavy metals displace essential elements, disturbing hormone synthesis and metabolic balance. Reproductive toxicity manifests through reduced fertility, impaired gametogenesis, and abnormal embryonic development, often mediated by epigenetic alterations. Neurological consequences include cognitive decline, behavioral changes, and neurodegenerative progression, driven by apoptosis, oxidative damage, and altered neurotransmission. Addressing these risks, recent advances in nanotechnology offer promising solutions for air quality management. Engineered nanomaterials including metal nanoparticles, carbon-based adsorbents, and metal organic frameworks demonstrate high efficiency in capturing and neutralizing airborne heavy metals. Nanostructured catalysts can transform toxic particulates into less harmful forms, while nanosensor platforms enable real-time monitoring at ultra-trace levels. This review synthesizes current evidence on the health impacts of heavy metal exposure, with particular emphasis on air pollution and emerging nanotechnology-based strategies for monitoring and remediation.

**Graphical Abstract:**

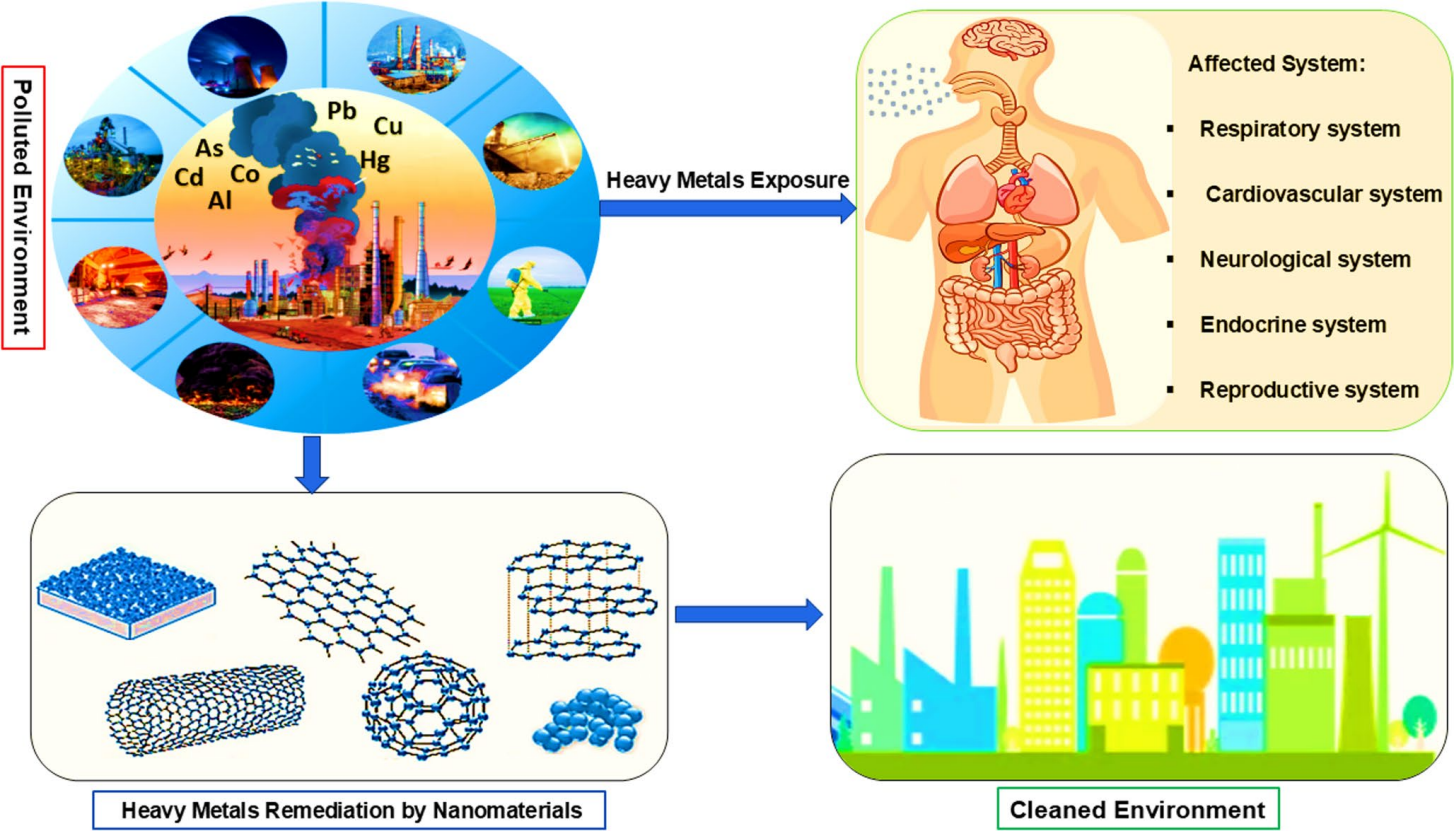

## Introduction

**Fig. 1:**
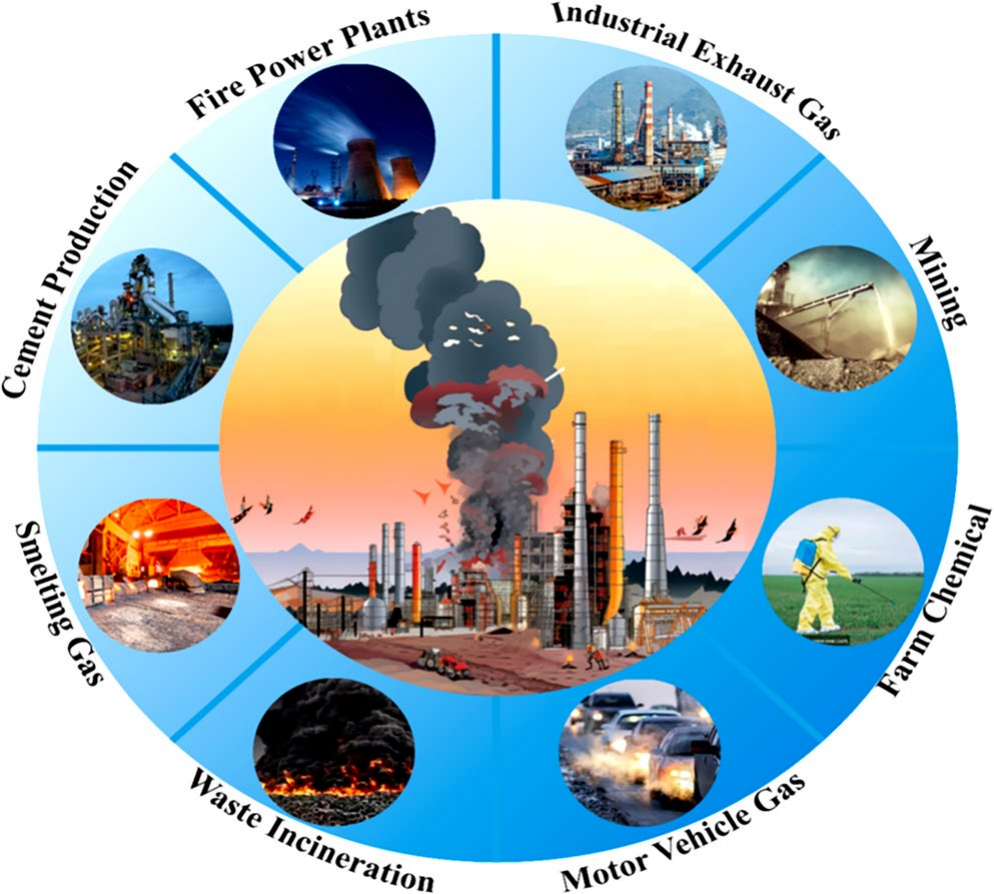
Sources of heavy metal air pollution [6]

Heavy metal air pollution, a critical public health concern, has intensified with industrialization, emitting toxic elements like arsenic (As), cadmium (Cd), cobalt (Co), copper (Cu), lead (Pb), mercury (Hg), and aluminum (Al). Industries such as power generation and metal smelting are major emission sources, with coal-fired plants notably contributing to flue gas emissions. High-temperature industrial activities also release heavy metals into the air, as shown in Fig. 1 [1]. The hazardous effects of airborne heavy metals extend beyond environmental degradation, posing severe risks to human health. Inhalation of contaminated particulates enables their direct entry into the respiratory system and bloodstream, leading to pulmonary fibrosis, chronic obstructive pulmonary disease (COPD), asthma, and reduced lung function [2]. Systemic distribution results in cardiovascular dysfunction, including endothelial damage, hypertension, and atherosclerosis, while renal exposure contributes to kidney damage and impaired detoxification. Endocrine toxicity arises when metals mimic or replace essential trace elements, disturbing hormone synthesis and metabolic regulation. Reproductive health is also compromised, as exposure reduces fertility, interferes with gametogenesis, and affects embryonic development. Neurological consequences are particularly profound, encompassing cognitive impairment, behavioral changes, and increased risk of neurodegenerative disorders [3]. These systemic health effects disproportionately affect vulnerable populations, particularly children, pregnant women, and immunocompromised individuals, due to their heightened biological susceptibility [4].

The rapid pace of industrialization and urbanization continues to escalate heavy metal emissions, emphasizing the urgent need for effective monitoring and remediation strategies. Conventional pollution control technologies and regulatory frameworks, while necessary, are often insufficient to address the complexity and persistence of heavy metal pollution. In this context, recent advances in nanotechnology have emerged as transformative solutions for air quality management. Engineered nanomaterials including metal nanoparticles, carbon-based adsorbents, and metal–organic frameworks exhibit remarkable capacity for capturing and neutralizing airborne heavy metals due to their high surface area, tunable porosity, and unique physicochemical properties. Furthermore, nanostructured catalysts facilitate the conversion of toxic particulates into less harmful forms, while Nanosensors platforms enable real-time monitoring at ultra-trace levels, thereby enhancing surveillance and supporting rapid interventions [5]. Collectively, these insights highlight the dual challenge posed by airborne heavy metals: their capacity to cause systemic toxicity and their resilience in the environment. Addressing these issues requires an integrated approach that combines toxicological understanding with innovative remediation strategies. Accordingly, this review first discusses the toxicological and systemic health impacts of airborne heavy metals, then explores their specific disease associations, and finally discusses emerging nanotechnology-based strategies for monitoring, detection, and remediation.

## Methodology

**Fig. 2:**
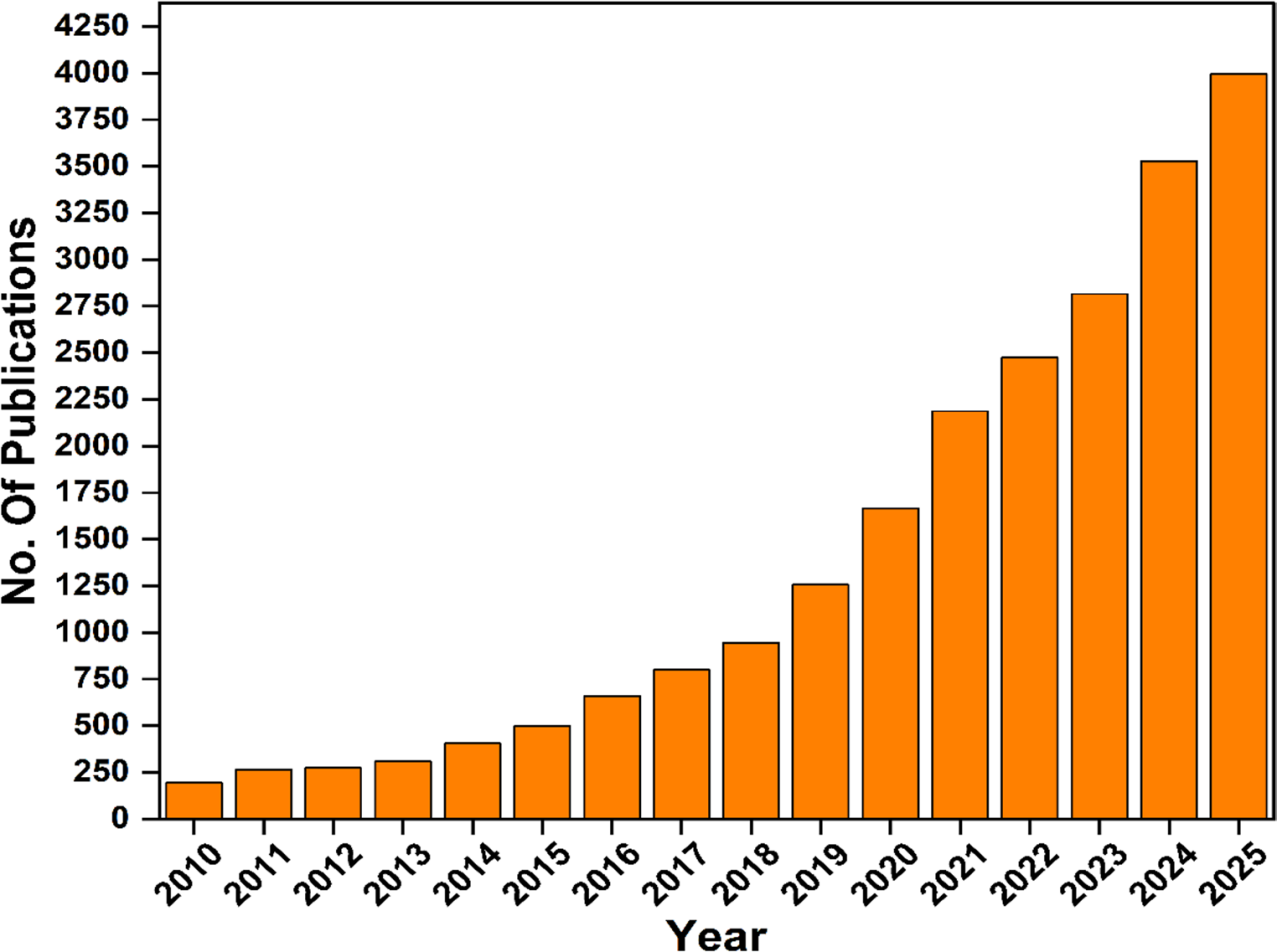
Number of publications on human health risks of heavy metal pollution and their remediation by using nanotechnology per year based on the Scopus database until 2025

The methodology of this review involved comprehensive literature searches across major academic databases, including PubMed, Scopus, Web of Science, and Google Scholar. The review focused on peer-reviewed studies published between 2010 and 2025, addressing established and emerging insights into heavy metals exposure and associated public health risks. Search terms were combined using Boolean operators and included keywords such as “Heavy metals”, “Air Pollution”, “Human health”, “Nanotechnology”, “Nanomaterials”. Initial screening involved titles and abstracts, followed by full-text evaluation. Studies were selected based on their relevance to the occurrence, sources, and types of heavy metals as well as their toxicological, physiological, or epidemiological effects. Emphasis was placed on research exploring both acute and chronic health outcomes associated with heavy metals exposure. Both original research and review articles were included, especially those discussing human exposure pathways and disease outcomes. Non-English language studies, articles without full text, and publications unrelated to heavy metals exposure or human health were excluded. Figure 2 showed that the interest of researchers in this area increased over time due to increasing sources of pollution nowadays because the technology.

## Overview of Heavy Metals

Heavy metals are harmful environmental contaminants with toxic effects on human health. Industrialization and related anthropogenic activities have increased exposure through air. The following subsections outline the major sources, exposure routes, health impacts, and regulatory limits of selected heavy metals of public health relevance.

### Arsenic (As)

Arsenic, is a toxic metalloid and recognized Group 1 human carcinogen [7]. Major sources of atmospheric As include mining, metal smelting, pesticide and herbicide use, wood preservation, glass production, and semiconductor industries [8]. Exposure occurs via inhalation of dust, ingestion of contaminated water and food, and dermal contact. Occupational Safety and Health Administration (OSHA) sets a permissible exposure limit (PEL) of 0.01 mg/m³ for arsenic dust while National Institute for Occupational Safety and Health (NIOSH) Recommended Exposure Limit (REL) of 0.002 mg/m^3^ [9]. All workers involved in copper slag processing approached or exceeded the OSHA PEL of 0.01 mg/m^3^ for arsenic, range: 0.009–0.018 mg/m^3^ [10]. It was reported that Arsenic concentrations in PM2.5 (2.87 ± 2.08 ng/m^3^) significant health concerns, which poses a substantial long-term carcinogenic threat [11]. Health impacts include skin lesions, neurological impairment, cardiovascular disease, and cancers of the lung, bladder, and kidney [12]. Mechanistically, As induces oxidative stress, disrupts endocrine and signaling pathways, and interferes with DNA repair. Its methylated metabolites, monomethyl arsenic (MMA) and dimethyl arsenic (DMA), may be more toxic than inorganic forms, linking toxicity closely to its metabolic processing [13].

### Cadmium (Cd)

Cadmium is a non-essential heavy metal released from fossil fuel burning, metal refining, battery production, pigments, plastics, and waste incineration [14, 15]. Tobacco smoke is another major source, as plants readily accumulate cadmium from soil [16]. Exposure occurs mainly through inhalation of polluted air or ingestion of contaminated food and water. OSHA sets a PEL of 0.05 mg/m³ for Cd dust while NIOSH set a REL of 0.2 mg/m³ [9]. It was reported that Cd dust level was 0.01 ± 0.006 which is below the recommended limit for occupational exposure set by NIOSH and OSHA, Nonetheless, prolonged low-level exposure induced DNA fragmentation and increased chromosomal aberrations in foundry workers [17]. Cd accumulates in the kidney and liver, with a long half-life, making chronic exposure especially dangerous. Toxic effects include renal dysfunction, bone demineralization, and respiratory diseases such as emphysema and lung cancer. Mechanistically, Cd induces oxidative stress, interferes with calcium and zinc metabolism, and disrupts mitochondrial function [14].

### Cobalt (Co)

Cobalt is a heavy metal present in air pollution, often bound to particulate matter, coming from sources of exposure include mining, smelting, battery manufacturing, and hard-metal industries [18]. Inhalation is a primary exposure route, leading to absorption and potential systemic harm. OSHA sets a PEL of 0.1 mg/m³, while NIOSH set a REL of 0.05 mg/m³ [9]. Co levels in hard metal industry were 0.0030 mg/m³ which were much below the recommended limit for occupational exposure. Nonetheless, prolonged low-level exposure entails severe adverse health effects, including lung cancer [19]. At toxic levels, Co causes occupational asthma, hard metal lung disease, dermatitis, cardiomyopathy, and cancer. Mechanistically, it generates reactive oxygen species (ROS), disrupts calcium homeostasis, and interferes with DNA repair processes, highlighting the fine line between its essentiality and toxicity.

### Copper (Cu)

Copper acts as a heavy metal pollutant in air, released from mining, smelting, welding, and significant traffic emissions, posing health risks like metal fume fever (MFF) [20]. OSHA and NIOSH recommend limits of 0.1 mg/m³ for copper dust [9]. While essential in trace amounts, excessive copper leads to gastrointestinal irritation, liver and kidney injury, and neurological disorders such as Wilson’s disease, Parkinson’s, and Alzheimer’s [21]. An acute self-limiting systemic inflammatory response to metal fume, were observed among three male workers exposed to copper-containing dust levels of 0.120 mg/m^3^ which is above OSHA and NIOSH recommended limits after lapping of copper plates [22]. Although modern regulations made cases of copper toxicity very rare in the developed world, it is still found in alarming amounts in developing countries like Nigeria, China, and Thailand where communities near factories are increasingly at risk [23, 24].

### Lead (Pb)

Lead has been widely used in fuels, paints, batteries, and pipes, remaining a persistent environmental pollutant despite restrictions [25, 26]. Major sources of atmospheric Pb include smelting, foundries, mining, and recycling of lead-based products. Inhalation of airborne particles and ingestion of contaminated food or water are the main exposure routes [27]. OSHA and NIOSH set limits of 0.05 mg/m³ for Pb dust [9]. Occupational exposure to Pb dust and fumes has a stimulatory effect on thyroid function as manifested by a significant increase in thyroid hormone levels [28]. It was reported that the average levels of Pb in the respiratory air in electrical solderers was 0.09 ± 0.01 mg/m^3^, which is higher than the recommended by OSHA and NIOSH [29]. Pb accumulates in bones and soft tissues, causing neurological deficits in children, renal impairment, anemia, and cardiovascular diseases. Mechanistically, Pb disrupts calcium signaling, inhibits heme synthesis, generates oxidative stress, and binds sulfhydryl groups, impairing protein function [30]. In pregnant mothers, Pb can pass through the placenta and accumulate in the fetus and may have adverse effects on ion transfer and can change the function of the membrane [31].

### Mercury (Hg)

Mercury is a highly toxic metal found in elemental, inorganic, and organic forms [32]. Exposure Sources include coal combustion, gold mining, chlor-alkali industries, and contaminated seafood [14]. Inhalation of vapor and ingestion of methylmercury are the main exposure routes. OSHA set PEL of 0.1 mg/m³ while NIOSH set a REL of 0.05 mg/m³ [33]. Hg targets the nervous, renal, immune, and respiratory systems. Toxicity mechanisms include binding to sulfhydryl (^−^SH) groups, inhibition of protein function, and induction of oxidative stress, leading to apoptosis and tissue damage [34, 35]. Long-term exposure to Hg vapor concentrations more than 0.02 mg/m^3^ may cause slight intoxication symptoms, while concentrations between 0.4 and 2 mg/m^3^ is expected to cause chronic Hg poisoning [36]. Dental workers are exposed to elevated levels of Hg vapor varied from 84.7 ± 18.67 to 609.3 ± 238.90 µg/m^3^ which were above the occupational exposure standards. The results of the biochemical parameters showed a significant increase in levels of cholesterol, aspartate aminotransferase (AST) and alanine aminotransferase (ALT) [37].

### Aluminum (Al)

Aluminium is considered a toxic metal in air pollution, contributing to poor air quality alongside other “heavy metals,” primarily entering the atmosphere from industrial sources (aluminum production, mining, smelting, coal burning, waste incineration) and vehicle exhaust [38]. Non-occupational exposure mainly arises from diet, food-contact materials, and drinking water, while inhalation is the main occupational pathway. OSHA sets a PEL of 15 mg/m³, while NIOSH sets a REL of 10 mg/m³ [9]. It was reported that Al dust level was 10.31 ± 3.73 mg/m^3^ in foundry workers which was above the recommended exposure limit set by NIOSH causing oxidative stress [3]. Al accumulates in the brain, bone, and kidneys, with reported effects including neurotoxicity, skeletal fragility, anemia Alzheimer’s disease and other neurodegenerative conditions [39]. Mechanistically, Al disrupts iron metabolism, induces oxidative stress, and promotes inflammatory responses. Table 1 summarizes a comparison of the major sources, exposure routes, environmental concentrations, and regulatory exposure limits of the heavy metals discussed above.

**Table 1:** Comparative overview of major airborne heavy metal, and regulatory limits

Heavy metal	Major sources	Primary exposure pathways	OSHA / NIOSH exposure limits (mg/m³)
As	Mining, smelting, pesticides, wood preservation, glass and semiconductor industries	Inhalation, ingestion	OSHA PEL: 0.01 NIOSH REL: 0.002
Cd	Fossil fuel combustion, batteries, pigments, plastics, waste incineration, tobacco smoke	Inhalation, ingestion	OSHA PEL: 0.05 NIOSH REL: 0.2
Co	Mining, smelting, battery manufacturing, hard-metal industries	Inhalation	OSHA PEL: 0.1 NIOSH REL: 0.05
Cu	Mining, smelting, welding, contaminated water systems	Inhalation, ingestion	OSHA PEL: 0.1 NIOSH REL: 0.1
Pb	Smelting, mining, recycling, batteries, legacy fuels and paints	Inhalation, ingestion	OSHA PEL: 0.05 NIOSH REL: 0.05
Hg	Coal combustion, gold mining, chlor-alkali industries, contaminated seafood	Inhalation, ingestion	OSHA PEL: 0.01 NIOSH REL: 0.01
Al	Mining, smelting, automotive and aerospace industries, food additives	Inhalation, ingestion	OSHA PEL:15, NIOSH REL:10

## Hazardous Effect of Heavy Metals Exposure on Human Health

Exposure to heavy metals poses significant health risks to human systems. These toxic elements can affect vital organs, disrupt metabolic processes, and interfere with cellular functions. Prolonged or elevated exposure often leads to respiratory, neurological, cardiovascular, endocrine, and reproductive disorders, causing several diseases.

### Respiratory System

**Fig. 3:**
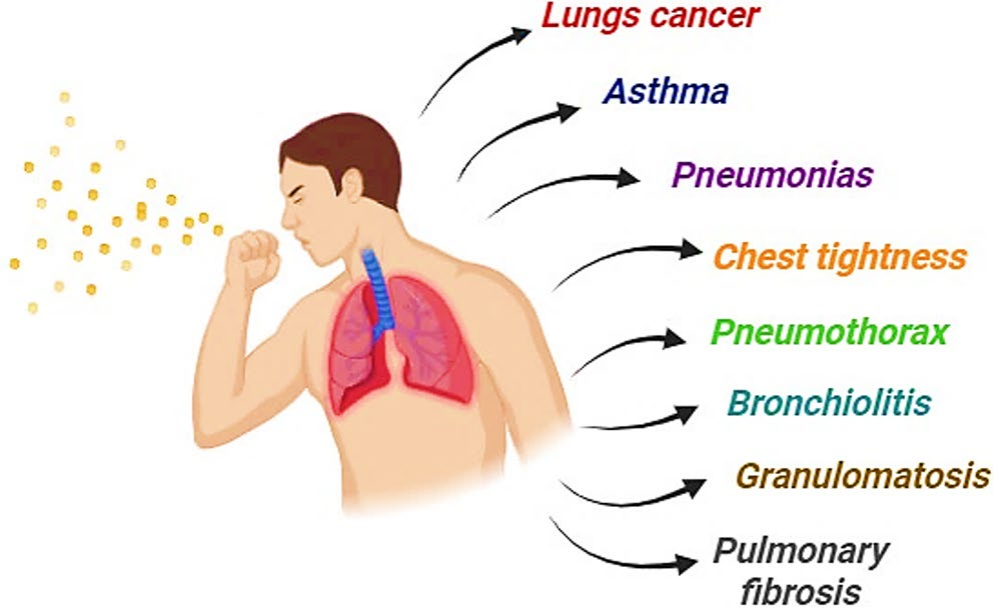
Effects of heavy metals exposure on respiratory system

Heavy metal exposure induces oxidative stress and inflammation in the respiratory system, leading to lung tissue damage. Chronic exposure increases the risk of respiratory diseases, including asthma, COPD, and lung cancer. These metals disrupt cellular function, impair immune responses, and precipitate and/or exacerbate pulmonary fibrosis and airway obstruction as shown in Fig. 3.

#### Effect of Arsenic Exposure

Occupational studies highlighted a significant risk of lung cancer associated with inhaling As, especially among miners and workers in metal smelting and refining industries [40]. Mechanistically, As exerts its toxicity by inhibiting repair enzymes containing dithiol groups, thereby increasing the production of ROS. These molecular alterations disrupt cellular integrity and promote malignant transformation. Clinical evidence also demonstrates negative interactions between pre-existing respiratory diseases and As exposure, with asthma patients experiencing particularly severe outcomes [41]. Furthermore, epidemiological data show that individuals with the highest urinary arsenic concentrations have a greater likelihood of developing asthma [42].

#### Effect of Cadmium Exposure

Acute inhalation of Cd produces irritation of the upper respiratory tract, manifested as coughing, sore throat, and dyspnea. The lungs absorb 40–60% of Cd in tobacco smoke, making smoking a major exposure source [43]. Prolonged Cd inhalation contributes to lung cancer and COPD through persistent inflammation involving macrophages, neutrophils, and proinflammatory cytokines [44]. In vitro studies demonstrate that Cd induces interleukin-6 (IL-6) and interleukin-8 (IL-8) secretion by primary human airway epithelial cells, promoting recruitment of inflammatory cells to sites of exposure [45]. In addition, autoimmune mechanisms leading to tissue damage have been described, impairing epithelial integrity and reducing pulmonary function [46]. Cd also directly targets airway and alveolar epithelial cells, resulting in cytotoxicity, airway obstruction, and asthma development [47].

#### Effect of Cobat Exposure

Cobalt exposure, especially in diamond and hard-metal industries, has been linked to hypersensitivity pneumonitis that may progress to pulmonary fibrosis. Clinical manifestations include cough, chest tightness, and bronchial asthma, often driven by specific immunological sensitization to cobalt [48]. Cobalt-induced cytotoxicity in lung cells further promotes DNA damage and tissue injury. Occupational studies reveal restrictive ventilatory impairment and reduced diffusing capacity, both associated with asthma, hard-metal lung disease, and increased risk of lung cancer [18, 49]. Additionally, dermal sensitization to cobalt has been shown to increase pulmonary susceptibility to inhaled cobalt particles [50].

#### Effect of Copper Exposure

Copper exposure contributes to airway irritation and lung cancer development through mechanisms involving cuproptosis and goblet cell hyperplasia, which may progress to malignant transformation [51]. Inhaled copper particles exhibit dose-dependent cytotoxicity, promoting the release of inflammatory mediators such as interleukin-4 (IL-4), interleukin-5 (IL-5), histamine, and prostaglandins. This cascade leads to airway hyper-responsiveness (AHR), inflammation, and cytotoxicity, particularly among welders and miners [52]. Epidemiological studies demonstrate widespread COPD and small airway disease (SAD) in exposed populations [53]. Associations between elevated serum copper and silicosis incidence have also been reported in workers, though the precise mechanisms remain unclear [54]. Beyond chronic obstructive conditions, copper exposure has been linked to nasal septal perforation, interstitial pulmonary fibrosis (IPF), and occupational pneumonia [55].

#### Effect of Lead Exposure

Although inhalation is a significant pathway of Pb absorption, its respiratory effects are less extensively studied compared to other metals. Nonetheless, occupational health studies indicate impaired lung function among lead-exposed workers [56]. Clinical observations reveal higher prevalence of respiratory symptoms and exacerbations in occupationally exposed populations compared to unexposed groups [57, 58]. Spirometry-based studies show negative correlations between blood lead levels and pulmonary function markers, including forced vital capacity (FVC) and forced expiratory volume in the first second (FEV1), both indicative of functional decline [59]. Animal models confirm pathological features characteristic of asthma, such as inflammatory infiltration, epithelial injury, mucus plugging, and vascular congestion, in lead-exposed lung tissues compared to controls [60]. Pb exposure is also associated with increased inflammatory markers including eosinophil peroxidase, nitric oxide, and endothelin-1, all of which are strongly linked to asthma and COPD pathophysiology [57]. Furthermore, Pb modifies immune responses by increasing antibody production, white blood cell (WBC) counts, and T-helper 2 (Th2) cell activity, further contributing to airway inflammation and hypersensitivity [61].

#### Effect of Mercury Exposure

Inhalation of Hg vapor results in rapid pulmonary absorption, followed by systemic distribution. Hg is oxidized into ionic forms that bind sulfur-containing amino acids, inhibiting sulfhydryl enzymes essential for cellular metabolism [62]. Elevated blood mercury levels are positively correlated with impaired lung function and increased airway inflammation, as reflected by elevated exhaled nitric oxide [63]. Pathological changes in lung tissue include alveolar collapse, thickening of alveolar walls, infiltration of inflammatory cells, and deposition of mercury pigments within the alveolar septa [64]. Histological findings often resemble acute respiratory distress syndrome (ARDS), with pneumocyte hyperplasia, intra-alveolar fibrin thrombosis, and hyaline membrane formation. Severe cases may progress to necrosis with complications such as interstitial emphysema, pneumomediastinum, and pneumothorax [65]. Pediatric studies report increased asthma risk in children with higher blood mercury levels, highlighting the risks of early-life exposure [63].

#### Effect of Aluminium Exposure

Occupational exposure to aluminum has been thoroughly investigated for its adverse respiratory outcomes. Workers exposed to high levels of aluminum-containing fine particulate matter (PM2.5), including truck drivers and smelter workers, demonstrate reduced FEV1 and FVC [66]. Chronic inhalation of aluminum dust can lead to progressive pulmonary fibrosis, termed aluminosis, a condition documented over the past seven decades [67]. Other aluminum-related diseases include asthma, alveolar proteinosis, lung cancer, granulomatosis, and bronchiolitis. Acute exposures typically cause airway irritation, cough, and dyspnea, while chronic exposures induce oxidative stress and inflammation, promoting fibrosis and impaired gas exchange [68]. A cross-sectional study on potroom workers demonstrated higher prevalence of respiratory symptoms and COPD, with reduced lung function associated with prolonged exposure [69]. Experimental findings further support aluminum’s pro-inflammatory role, with significant increases in sputum neutrophils and interleukin-8 protein levels within 24 hrs of exposure, confirming its contribution to airway inflammation and immune activation [70].

### Cardiovascular System

**Fig. 4:**
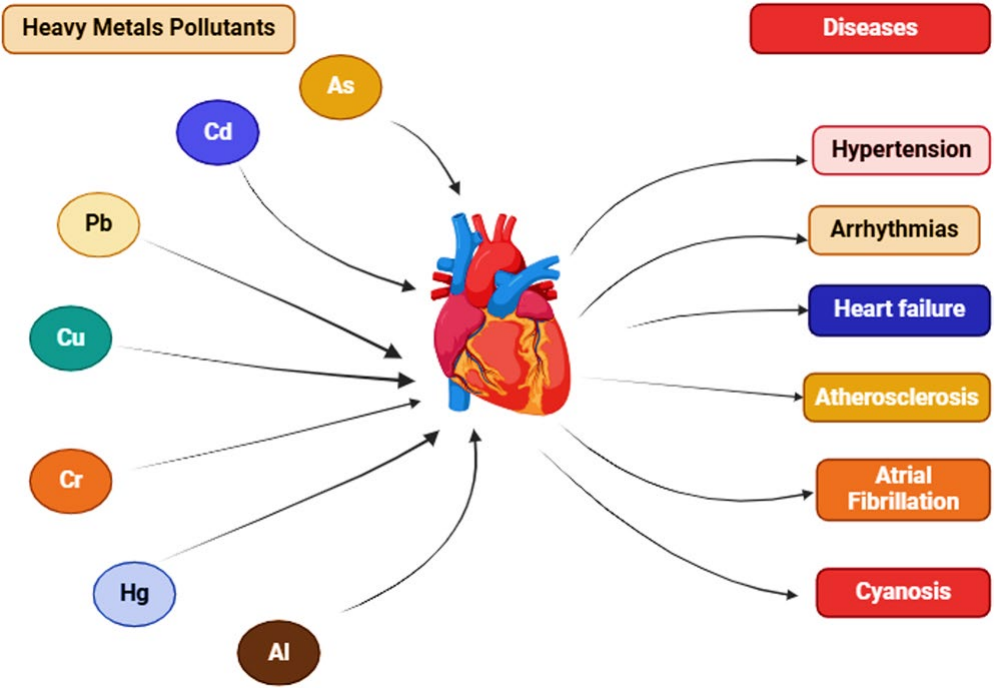
Effect of heavy metals exposure on cardiovascular system

Heavy metal exposure disrupts cardiovascular function by inducing oxidative stress, endothelial dysfunction, and inflammation. Chronic exposure increases the risk of hypertension, atrial fibrillation, atherosclerosis, and myocardial infarction as shown in Fig. 4.

#### Effect of Arsenic Exposure

Arsenic exposure has been strongly linked to cardiovascular alterations, with urinary arsenic levels positively correlated with carotid intima-media thickness (IMT), a marker of subclinical atherosclerosis [71]. Experimental studies show that in utero and early postnatal sodium arsenite exposure significantly accelerates atherosclerotic lesion development, highlighting risks even from early-life exposure [72]. Humans appear particularly vulnerable due to differences in arsenic methylation capacity compared with other species. Clinically, arsenic exposure has been associated with increased blood pressure and a higher prevalence of hypertension. Mechanistically, arsenic induces oxidative stress and endothelial dysfunction by decreasing nitric oxide (NO) bioavailability and impairing vasodilation. These changes stimulate vascular smooth muscle cell (VSMC) hypertrophy and hyperplasia, promoting vascular stiffness and remodeling. Furthermore, arsenic metabolites trigger apoptosis through ROS-dependent pathways and mitochondrial damage [73, 74]. Arsenic also acts synergistically with other metals, such as cadmium and titanium, to exacerbate atherosclerosis, while its direct effects on endothelial cells, smooth muscle cells, macrophages, and platelets contribute to peripheral vascular disease [75].

#### Effect of Cadmium Exposure

Cadmium exposure is considered an independent risk factor for cardiovascular disease. Even low levels impair cardiovascular health by altering cardiomyocyte formation and targeting renal proximal tubules, especially the S1 segment, thereby increasing chronic kidney disease risk, a known cardiovascular comorbidity [76]. Cd compromises endothelial integrity by inducing endothelial cell death and widening intercellular gaps, allowing metal accumulation within vascular walls. This promotes chronic inflammation through cytokines such as IL-6, IL-8, IL-1β, and tumor necrosis factor-alpha (TNF-α), which are elevated in cadmium-exposed individuals [77]. In cardiomyocytes, Cd disrupts calcium signaling, leading to impaired contractility and arrhythmogenic potential [78]. Long-term exposure raises total cholesterol and reduces NO bioavailability, compounding endothelial dysfunction and vascular injury [79]. Together, these mechanisms contribute to hypertension, cardiac dysfunction, and accelerated atherosclerosis.

#### Effect of Cobalt Exposure

High levels of cobalt exposure have been implicated in significant cardiovascular dysfunction. Clinical cases report rapidly progressive cobalt-induced cardiomyopathy, characterized by systolic dysfunction, heart failure, hypotension, cyanosis, and pericardial effusion, often accompanied by polycythemia and low-voltage electrocardiograms [80, 81]. Echocardiographic findings include left ventricular hypertrophy, biventricular dysfunction, and pericardial effusion [82]. Mechanistically, cobalt competes with essential cations such as magnesium and calcium, inhibiting key metabolic enzymes involved in the Krebs cycle, fatty acid metabolism, and electron transport [83]. This metabolic disruption reduces high-energy phosphate utilization and leads to myocardial insufficiency. Clinically, the result is myocardial weakness, ventricular dilatation, and secondary parietal thrombosis, all of which are characteristic of cobalt-related cardiotoxicity [84].

#### Effect of Copper Exposure

Copper exposure has been associated with cardiovascular events including hypertension, arrhythmias, and heart failure. Elevated Cu promotes low-density lipoprotein (LDL) oxidation, which accelerates atherogenesis and prothrombotic activity [85]. Homocysteine, an atherogenic amino acid, further enhances copper- and iron-dependent LDL oxidation, intensifying endothelial injury [86]. Epidemiological studies report higher risks of hypertension among copper-exposed individuals [24]. Occupational studies, particularly in automobile technicians, demonstrated prolonged prothrombin time (PT) and activated partial thromboplastin time (aPTT), indicating impaired coagulation pathways associated with copper exposure .Increased stroke incidence has also been documented in individuals with long-term copper exposure, further underscoring its cardiovascular burden [87].

#### Effect of Lead Exposure

Lead exposure has consistently been associated with hypertension and elevated blood pressure in both animal models and human populations [57]. Mechanistically, Pb contributes to cardiovascular dysfunction by enhancing inflammation, increasing ROS, and impairing DNA and membrane integrity [57, 88]. It also reduces antioxidant defense by inhibiting enzymes such as selenium-dependent glutathione peroxidase and copper-dependent superoxide dismutase, resulting in lipid peroxidation and endothelial damage [89]. Renal effects of lead, particularly through activation of the renin-angiotensin system, further contribute to hypertension and vascular injury [57, 89]. In addition, lead-induced anemia represents an indirect cardiovascular risk factor, as the metal inhibits delta-aminolaevulinic acid dehydratase, a key enzyme in heme synthesis, thereby disrupting red blood cell production [90]. Collectively, these alterations highlight lead’s role in hypertension and associated cardiovascular diseases.

#### Effect of Mercury Exposure

Chronic Hg exposure is strongly associated with cardiovascular disease and mortality. Clinical evidence links Hg to ischemic heart disease, coronary artery disease (CAD), angina, myocardial infarction, and arrhythmias [91]. A major source is dietary, especially from frequent consumption of large predatory fish [92]. Mechanistically, Hg promotes oxidative stress, endothelial dysfunction, and mitochondrial damage, while also contributing to dyslipidemia, thrombosis, and vascular inflammation [93]. Mercury-induced endothelial dysfunction narrows vascular lumens, increases blood pressure, and promotes proinflammatory cascades [94]. At the cellular level, Hg ions interfere with excitation-contraction coupling by competitively inhibiting calcium-troponin C interactions, leading to cardiomyocyte injury [95]. Furthermore, mercury enhances platelet aggregation and thrombus formation, raising risks of myocardial infarction and stroke [93]. Competition with iron for hemoglobin binding also contributes to hemolytic and aplastic anemia, conditions that may exacerbate cardiovascular dysfunction [96].

#### Effect of Aluminium Exposure

Aluminium exposure exerts adverse cardiovascular effects primarily through oxidative stress, inflammation, and apoptosis. It depletes reduced glutathione (GSH), inhibits antioxidant enzymes, and upregulates pro-oxidant systems such as NADPH oxidase and inducible nitric oxide synthase (iNOS) in cardiac and vascular cells [97]. These disruptions compromise vascular homeostasis and promote endothelial dysfunction. Experimental evidence from PC12 cell models shows that aluminum downregulates antioxidant defense pathways, including heme oxygenase-1 (HO-1) and nuclear factor erythroid 2–related factor 2 (Nrf2), further exacerbating oxidative injury [98]. These findings suggest that prolonged aluminum exposure can impair vascular resilience, contribute to hypertension, and increase susceptibility to cardiovascular disease.

### Neurological System

**Fig. 5:**
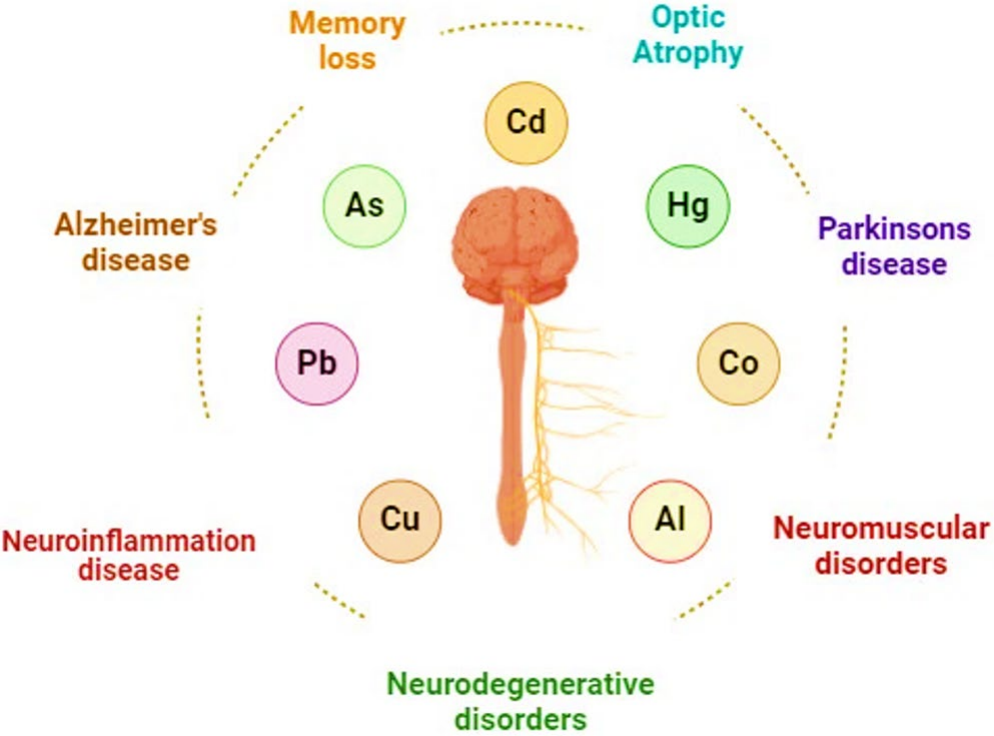
Effect of heavy metals exposure on neurological system

Heavy metal exposure causes neurotoxicity by inducing oxidative stress, neuroinflammation, and neurotransmitter imbalance. Chronic exposure leads to cognitive impairment, neurodegenerative diseases, and developmental deficits. These metals disrupt neuronal signaling, impair synaptic plasticity, and contribute to disorders like Alzheimer’s and Parkinson’s disease as shown in Fig. 5.

#### Effect of Arsenic Exposure

Arsenic can cross the blood–brain barrier and accumulating in brain tissue, leading to both peripheral and central neurological effects. Occupational exposure to inorganic arsenic has been associated with peripheral neuropathy, presenting with fatigue, pain, and paraesthesia in the extremities [99]. Electrophysiological studies reveal slowed sensory conduction velocities and decreased amplitudes in peripheral nerves. Animal experiments have demonstrated that arsenic exposure induces anxiety-like behaviors, impairs memory, and reduces locomotor activity. These functional deficits correlate with reduced Brain-Derived Neurotrophic Factor (BDNF) and N-methyl-D-aspartate receptor subunit NR2B in the hippocampus, alongside downregulation of estrogen receptor alpha (ERα). Antioxidant intervention with resveratrol has been shown to mitigate some of these effects, suggesting that arsenic disrupts neuronal homeostasis through oxidative stress and dysregulation of signaling pathways [100].

#### Effect of Cadmium Exposure

Cadmium exposure poses significant risks to the nervous system, particularly through its accumulation in choroid plexus, where it can cross into cerebrospinal fluid [101]. Its neurotoxicity is primarily mediated by oxidative stress, which damages neuronal membranes and reduces acetylcholinesterase activity, impairing cholinergic transmission [102]. Long-term cadmium exposure alters the microglial cells, the central nervous system’s resident immune cells, and contributes to neuroinflammation which can be linked to neurodegenerative disorders. Moreover, Cd also affects neurotransmitters including dopamine, serotonin, and glutamate. Hence, it can affect neuronal interaction and contribute to change of behavior and thought processes [103]. It can also interact with neuronal DNA, causing oxidative destruction of the strands or alterations in their sequence. This genotoxicity has the potential to disrupt neuronal function and contribute to the development of neurodegenerative changes [103].

#### Effect of Cobalt Exposure

Cobalt exposure is highly damaging to the eyes and ears through the production of ROS. In the eyes, cobalt exposure has been reported to cause bilateral optic atrophy and retinopathy [104]. This happens because cobalt reduces or blocks the receptive field surround of retinal neurons [105]. Cobalt was also linked to increased incidence of glaucoma by causing axonal edema and thinning of the myelin sheath resulting in progressive damage to the retinal ganglion cells and their axons [106]. It ultimately ends in irreversible or reversible visual loss, optic neuropathy, and atrophy. The auditory damage caused by cobalt is manifested by bilateral nerve deafness, which is characterized by damage of the hair cells, the peripheral auditory nerve fibers, and the spiral ganglion neurons [107].

#### Effect Copper Exposure

Copper accumulation in neural tissue has been associated with neurodegenerative processes. Elevated cerebrospinal fluid copper levels are found in copper storage diseases, which often present with neurological manifestations. Excess copper contributes to Alzheimer’s disease by binding amyloid-beta (Aβ), promoting its aggregation, and inducing oxidative stress [108, 109]. Furthermore, it is suggested that polymorphisms in the ATP7B gene, which plays a crucial role in copper balance, increase the risk of developing AD. Cu has a unique role in the pathophysiology of PD, as it aggregates in the substantia nigra, along with other metals, causing neurodegeneration that is driven by interactions between these metals and cellular proteins. These pathological interactions lead to protein aggregation and initiate a self-perpetuating cycle of mitochondrial dysfunction, ATP depletion, and the induction of inflammatory, oxidative, and nitrosative stress, ultimately culminating in cell death [110].

#### Effect of Lead Exposure

Lead exposure has both acute and chronic neurological consequences, with bone serving as a reservoir that sustains long-term release into circulation [111]. This gradual release of Pb is particularly concerning for children, as early exposure can manifest as neurological deficits later in adulthood [112, 113]. Pb mimics calcium, allowing it to enter neurons through voltage-gated calcium channels and disrupt neurotransmitter release, particularly gamma-aminobutyric acid (GABA) and glutamate [114]. The inhibition of Sirtuin 1 (SIRT1), a protein critical for regulating AMP-activated protein kinase (AMPK) was also linked to lead toxicity-induced apoptosis [112]. As with other metals, oxidative stress and inflammation have been extensively studied as contributing factors to neuronal and non-neuronal damage [57].

#### Effect of Mercury Exposure

Mercury is a potent neurotoxin, with methylmercury posing the greatest risk due to its ability to cross the blood–brain barrier. Prenatal exposure disrupts neuronal migration and differentiation, leading to long-term deficits in cognition, language, and motor skills [115]. In adults, chronic mercury exposure has been associated with tremors, memory loss, and neuromuscular dysfunction [116]. Mercury exerts its toxic effects on specific brain regions, notably the basal ganglia and cerebellum, exacerbating motor deficits [117]. Thus, Hg is involved in the pathogenesis of conditions like Alzheimer’s disease and Parkinson’s disease [118]. At the cellular level, mercury inhibits the mitochondria ATP synthesis leading to the loss of dopamine neurons in the substantia nigra [119]. Chronic exposure to mercury interferes with the polymerization of brain tubulin, crucial for microtubule formation and neurotransmitter transport in brain tissue. This disruption leads to cellular dysfunction, contributing to memory loss, difficulty in concentration, and potentially stimulating Tau protein hyperphosphorylation associated with Alzheimer’s disease [118]. Mercury-induced pathologies are common among individuals formerly employed in fluorescent lamp factories, artisanal and small-scale gold mining, and chemical manufacturing sectors [120].

#### Effect of Aluminium Exposure

Aluminium is recognized as a neurotoxin capable of causing substantial cognitive impairment and memory deficits, for example diminished auditory, visual, and working memory performance. Occupational exposure and toxicity were observed in inert gas metal welders and foundry workers [121]. Additionally, Al exposure affects executive and visuospatial functions, particularly the ability to identify spatial positions. Al neurotoxicity is also associated with neurodegenerative diseases such as familial Alzheimer’s disease, epilepsy, and Parkinson’s disease. This association was linked to accumulation and misfolding of amyloid proteins and hyperphosphorylation of specific tau proteins such as p-tau181 and p-tau231 which is central to neurofibrillary tangle formation [122]. Moreover, Al exposure was related to cytoskeletal proteins’ defects. Significant aggregation and disruption of cytoskeletal proteins in various brain regions, such as cerebral cortex, corpus striatum and hippocampus were reported [123].

### Endocrine and Reproductive System

**Fig. 6:**
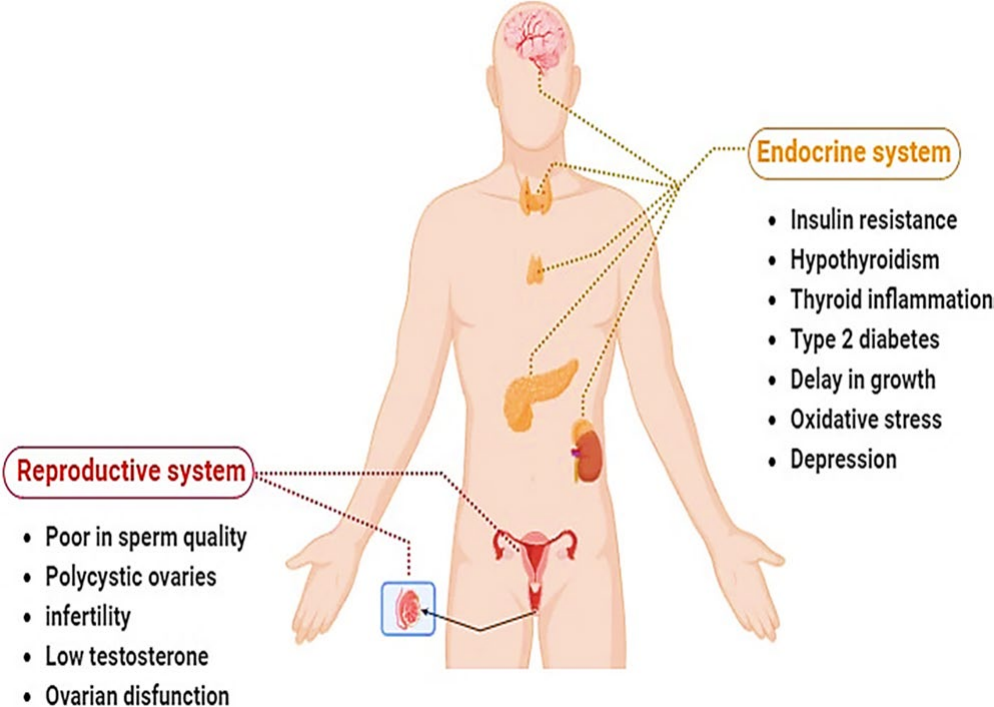
Effect of heavy metals exposure on endocrine and reproductive system

Heavy metal exposure disrupts endocrine signaling by interfering with hormone production, receptor binding, and metabolic regulation. This leads to thyroid dysfunction, metabolic diseases, reproductive dysfunction, infertility, menstrual irregularities, and developmental abnormalities. Chronic exposure alters thyroid, adrenal, and gonadal function, increasing the risk of hormonal imbalances and pregnancy complications as shown in Fig. 6.

#### Effect of Arsenic Exposure

Arsenic acts as an endocrine disruptor, as evidenced by in vitro activation of steroid hormone receptors. Specifically, arsenic exposure increases the wet weight of certain endocrine organs, such as the anterior pituitary gland and uterus. It also enhances the expression of the soluble guanylyl cyclase α1 subunit, linked to hormone-dependent tumor progression, and mildly reduces luteinizing hormone (LH) synthesis and release [124]. Chronic arsenic exposure leads to genotoxic damage and altered DNA methylation patterns that can be transmitted to offspring (transgenerational genotoxicity), and potentially linked to reproductive defects [125]. Inflammatory responses marked by increased IL-6, TNF-α, and cytokines such as IL-2, IL-5, IL-10, IFN-γ, and GM-CSF are also observed, indicating immune disruption. These immunological and epigenetic effects contribute to conditions including islet cell damage, diabetes mellitus, hyperuricemia, and peripheral arterial occlusive disease [126].

#### Effect of Cadmium Exposure

Cadmium disrupts several endocrine pathways, with the thyroid being one of the most affected organs. In animal studies, cadmium sulfate administration induced mitochondrial and endoplasmic reticulum damage in thyroid follicular epithelial cells and suppressed 5′-monodeiodinase activity through sulfhydryl group chelation [127]. Moreover, Cd interferes with pancreatic beta cells through oxidative stress and inhibition of zinc-containing enzymes that are used for insulin secretion, and this leads to insulin resistance and diabetes [128, 129]. Furthermore, Cd has disruptive effects on the production of gonadal steroids. It impairs the spermatogenic processes, which is manifested as a decrease in the concentration of sperm and abnormality of the sperm shape through its toxic effects on the seminiferous tubules, sertoli cells, and blood-testis barrier [130]. Additionally, Cd impairs binding of LH and FSH to their receptors and interferes with ovarian folliculogenesis, disrupting estrogen and progesterone production and leading to irregular menstrual cycles and reduced fertility [131].

#### Effect of Cobalt Exposure

Cobalt toxicity negatively affects several glands. Hypothyroidism and goitre have been shown in cases of cobalt exposure [132]. At the molecular level, cobalt inhibits tyrosine-iodinase, causes time-dependent tyrosine phosphorylation of mitogen-activated protein (MAP) kinase isoform ERK2, and inhibits thyroid-iodine uptake [133]. Furthermore, Co causes delay in growth via alteration of human growth hormone (GH) and increasing steroid metabolism [134]. It was reported that there is a greater risk for poor sperm quality among welders than among men not employed in welding [135]. Clinical reports in patients with metal-on-metal hip prostheses show increased sperm tail abnormalities, suggesting morphological disruption, although parameters such as motility and sperm count may remain unchanged [136]. A second study reported males with blood Co concentrations of 2–11 µg/L had no alterations in any of the sperm. These findings suggest that increased Co levels may influence the resulting sperm abnormalities [137].

#### Effect of Copper Exposure

Copper toxicity has been linked to several endocrine pathologies. There is an association between Cu intake and Type 2 DM with its complications, and hypothyroidism manifested by decrease T_3_ and T_4_ levels, and increase TSH levels [138, 139]. Cu toxicity is carcinogenic, where cells exposed to Cu experience a unique form of apoptosis [140, 141]. Furthermore, Cu can have a devastating effect on the reproductive health in males and females, being linked to erectile dysfunction and low testosterone in males while causing miscarriage, polycystic ovary syndrome (PCOS), impaired ovarian steroidogenesis and affected follicular development in females. At the mechanistic level, Cu has been linked to increase formation of advanced glycation end products, lipid peroxidation, proteotoxic effect and disruption of TCA [142].

#### Effect of Lead Exposure

Lead exposure was assessed in relation to functions of thyroid gland, reproductive organs, and pancreas with somewhat inconsistent results [143]. Few studies investigated the toxic effect of Pb exposure on pancreas. One such studies observed an association that does not prove a causal link, between increased blood Pb levels and diabetes. In addition, a correlation between Pb blood level and the incidence of non-alcoholic fatty liver disease, which in turn commonly co-exist with type 2 diabetes was reported [144]. Studies on the effect of Pb exposure on reproductive systems of both genders have been linked to altered hypothalamic-pituitary axis. In females, a link between women seeking in-vitro fertilization treatment and increased follicular fluid concentrations of lead was reported [145]. In males, high blood lead levels is associated with low sperm count, motility defects, and overall altered sperms morphology [146]. Mechanistically, Pb promotes oxidative stress and excessive ROS generation, which interferes with insulin signaling pathways, thereby contributing to insulin resistance and metabolic disease [144].

#### Effect of Mercury Exposure

Mercury interferes with endocrine and reproductive functions by disrupting multiple hormonal pathways. Even at low levels, it reduces hormone-receptor binding and inhibits enzymes involved in biosynthesis of insulin, estrogen, testosterone, and adrenaline [147]. The thyroid gland is a primary target, with both inorganic Hg and methylmercury inhibiting thyroglobulin iodination and thyroid hormone synthesis, leading to hypothyroidism, thyroid inflammation, and depressive symptoms [96]. Similar to its effects on the thyroid, Hg can affect the pancreas by binding to insulin’s sulfur-binding sites, disrupting normal biological function and causing dysregulation of blood glucose levels, leading to diabetes [148]. In the adrenal cortex, Hg reduces corticosterone production, causing compensatory increases in ACTH and adrenal hyperplasia. In reproductive health, mercury disrupts circulating levels of FSH, LH, estrogen, and progesterone, leading to ovarian dysfunction, irregular menstruation, premature menopause, and infertility in females. In males, Hg exposure results in DNA-damaged sperm, abnormal morphology, reduced motility, and erectile dysfunction, partly due to testicular accumulation [96].

#### Effect of Aluminium Exposure

Aluminium exposure impairs multiple endocrine glands, including the thyroid, pancreas, adrenal glands, and gonads. In the thyroid, Al inhibits thyroid peroxidase and iodide uptake, resulting in reduced T_3_ and T_4_ levels and increased TSH, consistent with hypothyroidism [149]. In the pancreas, Al accumulates in beta cells, induces oxidative stress, and impairs insulin synthesis and secretion, contributing to hyperglycemia and insulin resistance [150]. Adrenal toxicity occurs through inhibition of steroidogenic enzymes and disruption of the HPA axis, leading to reduced corticosterone levels and degeneration of adrenal tissue. Reproductive toxicity is also evident, with Al exposure lowering FSH, LH, and testosterone, alongside reduced sperm count and quality [151]. Nitric oxide elevation facilitates Al accumulation in the testes, where it penetrates the blood-testis barrier, damages Sertoli and germ cells, and interferes with calcium-mediated signaling essential for spermatogenesis. These effects collectively impair fertility and hormonal balance [152, 153]. Hazardous effects of heavy metals on various body systems are summarized below in Table 2.

**Table 2:** Effect of heavy metals exposure on human health and related diseases

Metal	Affected Organs/System	Health Effects	Ref.
Arsenic	Skin	Non melanoma Skin Cancer	[8]
	Lung	Cellular damage and oxidative stress, Asthma.	[41]
	Heart	Atherosclerosis, increased systolic and diastolic blood pressure, Hypertension.	[75]
	Neurological System	Extremity fatigue, extremity pain, paraesthesia in the lower extremities, and neuropathy	[154]
	Reproductive System	Abnormal morphology of ovaries and testicles, and transgenerational genotoxicity, reproductive defects	[155]
Copper	Neurological System	Alzheimer’s dementia, apoptotic death of neurons and astrocytes	[109]
	Heart	Hypertension, arrhythmias, and atrial fibrillation.	[24]
	Lung	Interstitial pulmonary fibrosis, nasal septal perforation, pneumonias	[55]
	Liver	Wilsons disease, cell injury via oxidative stress, hepatic damage	[156]
	Reproductive System	Polycystic ovary syndrome, erectile dysfunction, and low testosterone in males.	[142]
Cadmium	Lung	Chronic obstructive pulmonary disease, lung cancer.	[45]
	Heart	Coronary artery disease, peripheral artery disease, carotid artery disease	[157]
	Endocrine System	Insulin resistance, diabetes, oxidative stress in the endocrine glands	[128]
	Bone	Skeletal damage, itai-itai disease (a combination of osteomalacia and osteoporosis).	[158]
	Reproductive System	Irregular menstrual cycles, and low fertility rates	[131]
Cobalt	Lung	Pulmonary fibrosis, cough, chest tightness, and Bronchial asthma, increased risk of cancer by DNA breakage	[159]
	Heart	Heart failure, hypotension, cyanosis, pericardial effusion, fatigue, weakness, and polycythaemia	[81]
	Neurological System	Bilateral optic atrophy, retinopathy, bilateral nerve deafness, and sensory-motor polyneuropathy	[104]
	Endocrine System	Hypothyroidism and it is a prime cause of goitre, increased steroid metabolism so this causes a delay in growth	[134]
	Reproductive System	Growth delay, poor sperm quality, abnormality in sperm parameters	[137]
Lead	Lung	Asthma and Chronic obstructive pulmonary disease, congestion, epithelial injury, and mucus plug.	[60]
	Heart	Anemia, hypertension and an increase in both systolic and diastolic blood pressure.	[57]
	Brain	Cellular degradation and death, oxidative stress and inflammation.	[112]
	Endocrine System	Insulin resistance, type 2 diabetes, Non-alcoholic fatty liver disease	[144]
	Reproductive System	Reduced sperm count, motility, and overall altered morphology. affected the release of gonadotropic hormones in males	[146]
Mercury	Lung	Interstitial emphysema, pneumomediastinum and pneumothorax	[65]
	Heart	Mortality, and coronary artery disease. atherosclerosis, angina, heart attacks, and heart failure.	[91]
	Brain	Tremors, insomnia, memory loss, and neuromuscular effects.	[160]
	Endocrine System	Hypothyroidism, thyroid inflammation, depression and diabetes	[96, 161]
	Reproductive System	Erectile dysfunction, infertility in men and women, ovarian dysfunction	[96]
Aluminum	Respiratory System	Fibrosis, asthma, alveolar proteinosis, lung cancer, granulomatosis, and bronchiolitis.	[67]
	Cardiovascular System	Oxidative stress, apoptosis, and inflammation	[97]
	Brain	Familial Alzheimer’s disease, epilepsy, Parkinson’s disease	[122]
	Reproductive System	Testicular dysfunction, germ cell damage	[153]
	Breast	Breast cancer, proliferative benign breast disease	[162]

## Conventional Monitoring and Remediation of Heavy Metals

Traditional air monitoring uses high-volume air samplers, filter-based particulate collection, and cascade impactors [163]. The resulting samples are analyzed with laboratory methods such as atomic absorption spectroscopy (AAS) and inductively coupled plasma mass spectrometry (ICP-MS). These techniques yield precise data on metal concentrations and speciation. However, they are mainly costly, and time-consuming. This restricts their ability to track temporal variability and transient emission events. Delayed data and limited spatial resolution further hinder their effectiveness for real-time exposure assessment and prompt regulatory action [164]. Conventional remediation methods for heavy metals mainly use physical separation and bulk adsorption. Technologies such as electrostatic precipitators, fabric filters, and moist scrubbers are widely employed in industrial settings. These systems help control particulate-bound metal emissions [165]. They work well for coarse particles but often struggle with ultrafine particles and volatile metals like elemental mercury. Chemical scrubbing and precipitation techniques can improve removal. However, they also cause high energy use, secondary waste, corrosion, and complex operations. Bulk adsorbents, like activated carbon and mineral-based sorbents, are used to remove gaseous heavy metals from flue gases. These materials are cost-effective and scalable. However, they often lack selectivity, act slowly, and exhibit poor performance at low concentrations, especially in complex mixtures. Regenerating and reusing conventional sorbents is also difficult. This increases operational costs and environmental impact. Together, these limitations show major gaps in traditional monitoring and remediation [166]. There is a lack of real-time detection and limited selectivity for certain metals. Systems are less effective for ultrafine and volatile contaminants, and sustainability is a concern. These challenges have led to the development of new material-based solutions. These offer better sensitivity, faster response, and multifunctionality. As a result, nanotechnology is being integrated into the detection and remediation of airborne heavy metals.

## Nanotechnology and Nanomaterials Used in Heavy Metal Remediation

Nanotechnology is presented as a remedial and monitoring strategy, employing engineered nanomaterials designed to detect, capture, or transform toxic metal species. In this section, heavy metals are discussed exclusively as airborne environmental contaminants responsible for adverse health and ecological effects. Although certain metal and metal oxide nanoparticles may exhibit intrinsic toxicity under uncontrolled exposure, their discussion here is limited to intentionally engineered systems applied under controlled conditions for pollution mitigation and sensing, rather than as environmental pollutants themselves.

### Synthesis of Nanoparticles

**Fig. 7:**
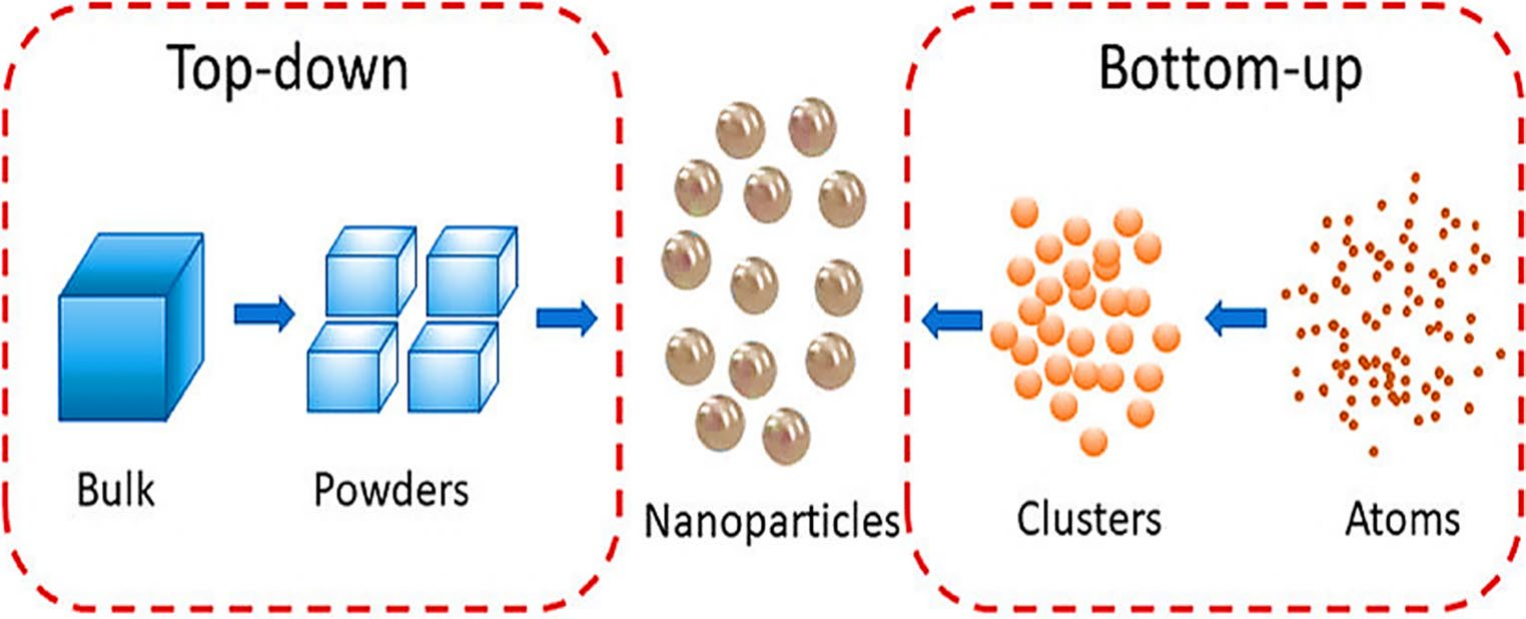
Scheme of Top-down and Bottom-up methods for nanoparticles synthesis [170]

The synthesis technique of nanomaterials plays a significant role in the final characteristics exhibited. Generally, the synthesis route of nanomaterials follows two ways; Top-down and Bottom-up approaches, as shown in Fig. 7. Top-down strategies, such as milling, grinding, and physical vapor deposition, reduce bulk materials into nanoscale structures but often result in broader particle size distributions [167]. Bottom-up methods, including sol–gel processing, hydrothermal synthesis, aerosol-assisted deposition, and chemical vapor deposition, construct nanoparticles from molecular precursors, allowing for precise control over particle morphology, porosity, and surface functionality [168]. More recently, green synthesis has emerged as a sustainable route for nanoparticle production. This method employs biological resources such as plants, algae, fungi, and bacteria to generate nanoparticles in a cost-effective, safe, and environmentally friendly manner. Plant-mediated synthesis has gained attention because it eliminates the need for microbial cultures and complex purification steps, yielding nanoparticles with biocompatible surface coatings [169].

### Classification of Nanomaterials Used in Heavy Metal Remediation

Nanomaterials employed for heavy metal monitoring and remediation can be broadly classified based on composition, structure, and functional properties. This classification helps clarify their role as engineered solutions rather than environmental contaminants.

#### Transition Metal Nanoparticle

Transition metal nanoparticles dispersed on porous supports have attracted considerable attention as efficient catalysts for the adsorption and reduction of airborne heavy metals [171]. Their nanoscale dimensions maximize active surface area and metal dispersion, while supports such as silica and activated carbon provide structural stability and prevent particle agglomeration [172]. Tuning parameters such as metal type, particle size, morphology, and support interaction allows optimization of catalytic selectivity for targeted heavy metal removal. Manganese nanoparticles (Mn NPs) display strong affinity for toxic metals. *Islam et al.* prepared mesoporous silica-encapsulated Mn NPs (Mn@mSiO₂, 10–30 nm) using a microemulsion method [173]. These catalysts achieved removal capacities of 193 mg/g for Pb (II) and 322 mg/g for Cd (II) from simulated flue gas. Iron nanoparticles are widely employed due to their high reactivity, low cost, and environmental compatibility [174]. Ultrafine Fe NPs (~ 3 nm) synthesized via hydrogen reduction efficiently captured Hg(II) from flue gas [175]. Their superior redox activity enabled conversion of Hg⁰ to Hg (II), which was rapidly adsorbed. These Fe NPs maintained > 90% mercury removal efficiency for over 100 h of operation. Cobalt nanoparticles exploit multiple oxidation states to promote reduction of Cr(VI) and As(V) [176]. Flower-like Co NPs synthesized in diethylene glycol and immobilized on biochar demonstrated a Cd(II) removal capacity of 124 mg/g [177]. Spectroscopic studies revealed partial reduction of Cd (II) to Cd (0) through electron transfer from Co (II), which was simultaneously oxidized to Co (III). Nickel nanoparticles are notable for their strong reducibility and catalytic activity [178]. Ni NPs anchored on nitrogen-doped graphene achieved 96.6% gas-phase Cr(VI) removal efficiency [179]. In situ DRIFTS and XANES confirmed that Cr (VI) was both adsorbed and reduced to Cr (III) on the Ni NP surface. Copper nanoparticles exhibit strong binding affinity for sulfur-containing species. Thiol-functionalized Cu NPs synthesized via NaBH₄ reduction removed > 99% of SO₂ and Hg(II) from simulated flue gas [180]. Their Hg (II) adsorption capacity was 10-fold higher than commercial CuO and five-fold higher than CuS. Zinc nanoparticles, which offer abundant Lewis acidic sites, preferentially bind soft heavy metals such as Hg(II) [181]. Porous ZnO-supported Zn NPs derived from a zinc–ethanolamine precursor achieved > 90% Hg⁰ capture from flue gas over 180 h of continuous operation [182].

#### Metal Oxide Nanomaterials

Metal oxide nanoparticles represent a key class of nanomaterials for the remediation of airborne heavy metals, offering a combination of adsorption, redox activity, and photocatalytic properties [183]. Semiconducting oxides such as titanium dioxide (TiO₂), zinc oxide (ZnO), iron oxides (Fe₃O₄, γ-Fe₂O₃), manganese oxides (MnOₓ), and tin dioxide (SnO₂) are particularly effective due to their ability to catalyze the oxidation of toxic metals like elemental mercury (Hg⁰) and arsenic (As(III)) under UV or solar irradiation, transforming them into less volatile and more easily captured species [184]. TiO₂ nanoparticles are widely studied for their strong photocatalytic activity, producing ROS that oxidize gaseous metals into stable oxides such as HgO [185]. Iron oxides provide an additional advantage through their magnetic properties, allowing easy recovery and reuse of the adsorbent, thereby minimizing secondary contamination. MnOₓ nanoparticles possess abundant surface redox sites that facilitate rapid oxidation of metals like arsenic and selenium, while copper oxides have demonstrated dual benefits, contributing to heavy metal capture and providing antimicrobial action in multipollutant environments [186]. Nanoscale structuring enhances the density of active sites, increases surface defects, and improves charge separation, collectively boosting catalytic efficiency.

#### Carbon-Based Nanomaterials

Carbon-based nanomaterials such as graphene and carbon nanotubes (CNTs) are highly effective for capturing airborne heavy metals because of their large surface area, tunable surface chemistry, and strong adsorption capacity [187]. Functionalization with oxygenated groups increases their affinity for toxic metal vapors like Hg⁰, Pb (II), and Cd (II), enabling rapid and selective removal from polluted air streams. Despite their efficiency, careful assessment of potential release, toxicity, and environmental persistence is needed for safe application. Activated carbon (AC), produced from coal, wood, or agricultural residues, is widely applied in air filtration systems for its high porosity and stability. It efficiently adsorbs heavy metal vapors in flue gas treatment and can be combined with catalytic coatings to enhance oxidation and capture efficiency [188].

##### Carbon Nanotubes

Carbon Nanotubes (CNTs) are cylindrical nanomaterials composed of rolled graphene layers, valued for their exceptional tensile strength, high electrical conductivity, and chemical stability [189]. Their extremely large specific surface area provides numerous active sites, enabling efficient adsorption of airborne heavy metals such as Pb (II), Hg (II), Cd (II), and As (III) emitted from industrial processes. Multi-walled carbon nanotubes (MWCNTs) are particularly attractive due to their higher surface area and electrical conductivity compared with single-walled CNTs, which enhances their adsorption capacity. Surface functionalization, such as plasma oxidation, introduces oxygenated groups that improve metal ion binding through electrostatic interactions and surface complexation. Composites of MWCNTs with metal oxides like Fe₃O₄ and MnOₓ have demonstrated synergistic performance, combining adsorption with catalytic oxidation of volatile metal species. Functionalized MWCNTs have been reported to be up to 20 times more effective in metal removal than pristine MWCNTs, making them highly promising for heavy metal remediation in polluted air [187].

##### Graphene Nanomaterials

Graphene, a single-atom-thick sheet of carbon arranged in a hexagonal lattice, has emerged as a highly efficient adsorbent for heavy metal remediation from aqueous systems. Its derivatives, including graphene oxide (GO) and reduced graphene oxide (rGO), exhibit exceptional sorption properties through mechanisms such as ion exchange, electrostatic interactions, and surface complexation. The abundance of oxygenated functional groups (e.g., hydroxyl, epoxy, and carboxyl) on GO significantly improves its affinity for metal ions. Graphene-based sorbents have demonstrated remarkable efficiency in capturing contaminants like Cr (VI), Pb (II), and Hg (II), and can be further modified to improve selectivity and adsorption performance [190, 191, 192]. Experimental studies report that GO can eliminate nearly 98% of Pb (II) from solutions in relatively short contact times, underscoring its rapid kinetics. Likewise, rGO shows outstanding uptake capacities for Ag⁺ and Cr species, highlighting its potential for practical remediation applications. The primary removal mechanisms electrostatic attraction, hydrogen bonding, and surface coordination collectively enable these materials to effectively bind and immobilize heavy metals from polluted water sources [190, 193].

#### Metal–Organic Frameworks (MOFs)

Metal–Organic Frameworks (MOFs) are an advanced class of porous nanomaterials with exceptional surface areas, often exceeding 7000 m²/g, making them highly effective for trapping airborne heavy metals [194]. They are composed of metal nodes coordinated with organic linkers, forming tunable porous networks. By modifying the metal clusters or organic ligands, MOFs can be engineered to selectively capture targeted heavy metal species from complex gaseous environments. MOFs synthesis methods categorized by reaction conditions are given in Table 3. Beyond adsorption, certain MOFs exhibit photocatalytic activity, enabling light-driven degradation or transformation of toxic metal pollutants. Their open metal sites and tailored pore functionalities promote strong coordination or chelation with heavy metals, enhancing removal efficiency. For instance, *Huang et al.*, developed a thiol-functionalized MOF, [Zn^2^ (atb)^2^(bpee)] (MOF-2), which achieved outstanding As(III) and As(V) capture from simulated flue gas, with capacities of 201 mg/g and 266 mg/g, respectively far surpassing conventional sorbents [195]. The presence of thiol groups facilitated strong As–S coordination, accounting for its superior performance. Photoactive MOFs containing Ti, Fe, or Zr clusters can produce reactive oxygen species under light irradiation, enabling in situ oxidation of volatile heavy metals. Ti-based MIL-125-NH₂, for example, completely oxidized toxic Hg⁰ vapor under visible light, converting it to Hg (II), which was subsequently immobilized within the MOF pores [196]. Similarly, functionalization with amine groups has proven effective for capturing anionic species such as Cr (VI) and As (V). *Li et al.*, reported an ethylenediamine-modified MOF that removed Cr (VI) with a capacity of 199 mg/g, simultaneously reducing it to the less toxic Cr(III) form [197].

**Table 3:** Synthesis methods and adsorption capacities of representative MOFs for heavy metal ion removal

MOFs	Method	Capacity	Target	Ref.
UiO-66-NH2	Solvothermal	198.7 mg/g	Pb (II)	[194]
Cu-BTC	Hydrothermal	167.2 mg/g	Hg (II)	[198]
Cd-MOFs	Microwave assisted	71.4 mg/g	Cr (VI)	[199]
Cu-BTC/GO	Hydrothermal	152.6 mg/g	Cd (II)	[200]
Fe-MIL-101	Solvothermal	198.4 mg/g	Cr (VI)	[201]

### Nanosensors and Smart Detection Tools

Nanosensors are a major advancement in air quality monitoring, providing continuous, highly sensitive detection of particulate matter, heavy metals, and toxic gases. Modern nanosensor systems can measure pollutants like PM, NOx, SO₂, CO, O₃, and Volatile Organic Compounds (VOCs) with high precision and fast response, enabling timely interventions. Increasingly, these systems are integrated into Internet of Things (IoT) platforms, allowing remote data acquisition, wireless communication, and real-time analytics for predictive modeling and adaptive pollution management. Different classes of nanomaterials are explored for sensor development, each offering unique mechanisms to enhance sensitivity and specificity.

#### Metal Oxide Nanostructures

Engineered synthesized Metal oxides NPs such as ZnO, TiO₂, SnO₂, and CeO₂ are widely used in optical and electrochemical sensors for gas sensing and environmental monitoring. TiO₂ nanomaterials exhibit strong gas adsorption properties, making them effective for detecting VOCs and air pollutants like NO₂ and O₃. The use of metal oxides highlights the value of inorganic nanostructures, but other nanomaterials, such as semiconductor quantum dots, offer complementary optical properties that expand detection possibilities [202].

#### Quantum Dots (QDs)

Quantum dots offer size-tunable emission and excellent photostability, making them ideal for fluorescence-based sensing of gases, pH, ions, and biomolecules. Silicon quantum dots are investigated for bioimaging applications and their implications for public health, while Carbon Quantum Dots (CQDs) provide a low-cost, sensitive, and non-invasive approach for metal ion detection and biosensing. While QDs excel in optical detection, carbon-based nanomaterials bring outstanding electrical conductivity and mechanical stability, which make them excellent electrode materials [203].

#### Carbon Nanomaterials

Carbon-based nanomaterials including CNTs, graphene, graphene oxide, carbon dots, and graphene quantum dots are extensively used as electrode materials due to their excellent electrical conductivity and surface reactivity. These features enable precise detection of heavy metal ions, gas pollutants, and biological analytes. To further improve sensor performance, researchers have turned to metallic nanoparticles, which can enhance signal strength and lower detection limits [204].

#### Metal Nanoparticles

Engineered Metal nanoparticles such as Au, Ag, Cu, Ni, Fe, and Zn are critical in developing highly sensitive Nanosensors. They are often used to modify electrodes and enhance detection limits. Bimetallic nanoparticles (e.g., Ag–Au) have been applied for detecting pollutants such as Cr (VI) in wastewater with improved sensitivity. Combining these nanomaterials into hybrid platforms can yield synergistic effects, producing sensors with even higher selectivity and responsiveness [205].

#### Hybrid Nanomaterials and MXenes

Hybrid nanomaterials combine metal oxides, carbon nanomaterials, or metal nanoparticles to achieve superior sensor performance. Functionalized MXenes are emerging as promising materials for VOCs detection and are easily integrated into IoT-based sensing devices. These material innovations collectively enable sensors that are not only more sensitive but also suitable for continuous and real-time monitoring [206].

## Environmental Safety and Risk Considerations

### Ecotoxicity and Environmental Concerns

While nanomaterials offer promising solutions for air pollution control, their release into the environment raises concerns about toxicity and long-term ecological impact. Their small size, high surface area, and reactivity allow them to interact with biological systems in ways not observed with bulk materials, potentially disrupting cellular processes in bacteria, plants, and animals. Nanoparticles may bioaccumulate within organisms and biomagnify along food chains, leading to elevated concentrations in predators and chronic ecological risks [207]. The environmental fate of nanomaterials depends on their mobility and persistence. Due to their nanoscale dimensions, nanoparticles can travel long distances in air, migrate through soil pores, and disperse in aquatic systems. Surface charge, aggregation behavior, and interactions with organic matter affect their bioavailability and potential for uptake by plants and organisms. Persistent nanomaterials that resist degradation can accumulate in soil and water, altering ecosystem functions and contaminating natural resources [208].

### Risk Assessment and Regulatory Frameworks

To minimize unintended impacts, robust risk assessment and regulatory measures must accompany the deployment of nanotechnology. Comprehensive risk assessment should consider nanomaterial composition, size, surface chemistry, environmental persistence, and potential exposure pathway [209]. Life cycle assessment (LCA) provides a framework for evaluating impacts from synthesis to disposal, ensuring sustainability across the material’s lifespan. Regulatory strategies should mandate standardized toxicity testing, clear labeling of nanomaterial-containing products, and safe disposal guidelines. Biomonitoring programs are essential to track nanoparticle concentrations in air, soil, and water and assess their impact on ecosystems and human health [210]. International harmonization of regulations can promote transparency, consistent safety standards, and responsible nanomaterial management across borders.

### Safety by Design and Mitigation Strategies

Mitigating environmental and health risks requires proactive approaches to control nanoparticle release during synthesis, application, and disposal [211]. Strategies include immobilizing nanomaterials in stable matrices (polymers, ceramics, or composites), developing retrievable magnetic nanoparticles, and designing recyclable or biodegradable nanomaterials to prevent uncontrolled accumulation. Sustainable production methods such as green synthesis using plants, microbes, or waste biomass reduce toxic byproducts and energy demand [212]. By integrating safe by design principles with comprehensive risk assessment and regulatory oversight, nanotechnology can be deployed for air pollution remediation while minimizing adverse ecological and human health impacts.

## Challenges and Future Prospects

Despite promising advances, several barriers hinder the large-scale application of nanotechnology for heavy metal air pollution control. Scaling up nanomaterial production remains difficult, as maintaining consistent size, morphology, and functionality is resource-intensive and often leads to performance variability. Integrating these materials into existing systems, such as filters and catalytic converters, requires additional engineering and regulatory approvals, delaying commercialization. Balancing performance with cost and sustainability is another key challenge, as many high-efficiency nanomaterials (e.g., noble metals) are expensive and environmentally costly to produce. Future efforts should emphasize green, low-energy synthesis methods, recyclable nanomaterials, and waste-derived resources to minimize secondary pollution. Nanosensors networks hold great promises for real-time air quality monitoring, but issues such as long-term stability, calibration, and data security must be resolved before widespread deployment. Energy-efficient, self-calibrating Nanosensors with standardized IoT communication protocols are needed for reliable operation. Looking ahead, integrating nanotechnology with artificial intelligence (AI), big data, and renewable energy could enable predictive pollution modeling, optimized remediation strategies, and autonomous, sustainable air purification systems. Achieving these goals will require interdisciplinary collaboration between researchers, engineers, policymakers, and industry stakeholders to align innovation with public health and environmental sustainability.

## Conclusion

Heavy metal air pollution remains a persistent global health threat due to its ability to bioaccumulate in human tissues and disrupt multiple organ systems through oxidative stress, chronic inflammation, and interference with cellular signaling. These mechanisms drive respiratory, cardiovascular, neurological, endocrine, and reproductive disorders, contributing to significant morbidity and mortality. Industrialization and urbanization continue to escalate heavy metal emissions, emphasizing the urgent need for comprehensive monitoring and remediation strategies. Recent advances in nanotechnology have revolutionized air quality management by providing efficient tools for monitoring, detection, and remediation of heavy metal pollution. Engineered nanomaterials including metal oxides, carbon nanostructures, quantum dots, metal nanoparticles, hybrid nanomaterials, and MXenes offer high surface area, tunable reactivity, and rapid response. Nanosensors integrated with IoT platforms enable continuous, real-time surveillance and data-driven interventions, while nanostructured adsorbents and catalysts capture, neutralize, or transform toxic particulates into less harmful forms, contributing to cleaner air. However, the use of nanotechnology must be carefully managed to avoid environmental and health risks. The mobility and persistence of nanomaterials raise concerns about unintended exposure, bioaccumulation, and secondary pollution. Safe by design approaches, green synthesis methods, life cycle assessment, and strong regulatory frameworks are essential to ensure sustainability. Moving forward, integrating advanced Nanosensors networks with responsible nanomaterial engineering and coordinated public health strategies will be critical to mitigating heavy metal emissions while protecting ecosystems and human health.

## Data Availability

No datasets were generated or analysed during the current study.
